# Correction: Freitas et al. Bacterial Cellulose/Tomato Puree Edible Films as Moisture Barrier Structures in Multicomponent Foods. *Foods* 2022, *11*, 2336

**DOI:** 10.3390/foods12061232

**Published:** 2023-03-14

**Authors:** John A. M. Freitas, Giovana M. N. Mendonça, Leticia B. Santos, Jovan D. Alonso, Juliana F. Mendes, Hernane S. Barud, Henriette M. C. Azeredo

**Affiliations:** 1 Graduate Program in Biotechnology, Federal University of São Carlos (UFSCar), São Carlos 13565-905, Brazil; 2 Graduate Program in Food, Nutrition and Food Engineering, UNESP—São Paulo State University, Araraquara 14800-903, Brazil; 3 Institute of Chemistry of Araraquara, UNESP—São Paulo State University, São Paulo 01049-010, Brazil; 4 Embrapa Instrumentation, São Carlos 13560-970, Brazil; 5 Biopolymers and Biomaterials Group, University of Araraquara (UNIARA), Araraquara 14801-340, Brazil

## Error in Table

In the original publication [1], there was a mistake in Table 2 as published. The numbers of NG and NFG rows were swapped in the last two columns. The corrected Table 2 appears below.

The authors state that the scientific conclusions are unaffected. This correction was approved by the Academic Editor. The original publication has also been updadated.

## Figures and Tables

**Table 2:** Results from sensory evaluation of nachos covered with guacamole (NG) or with film (film #3, produced by continuous casting) and guacamole (NFG).

Samples	Acceptance *	Number of Favorable Responses on Paired Preference Test	Number of Responses as “Crispier” ^‡^
NG	7.70 ± 1.36	23	26
NFG	8.03 ± 1.27	**57**	**52**

* Acceptance values on a 9-point structured scale (from 1—“extremely disliked” to 9—“extremely liked”); values presented as means ± standard deviations. ^‡^ Two panelists answered that they did not notice differences in crispiness between the samples. Bold values for the paired preference and crispiness comparison represent signiﬁcant preference/difference (values higher than 50, presented as the lowest required value for a signiﬁcant preference in a sensory panel with 80 panelists, according to Lawless and Heymann) [23].
